# Strengthening geospatial surveillance for malaria control and elimination in Indonesia: a meeting report

**DOI:** 10.1186/s12936-026-06184-5

**Published:** 2026-10-13

**Authors:** Abdillah Farkhan, Massaya Sirimatayanant, Iqbal R. F. Elyazar, Karina Dian Lestari, Fadjar S. M. Silalahi, Helen Dewi Prameswari, Hermawan Susanto, Era Susanti, Riskha Tiara Puspa Dewi, Dedy Supriyanto, Sri Budi Fajariyani, Made Bhagaskara Hadi Prawira, Septian Suhandono, Richard James Maude, Chawarat Rotejanaprasert

**Affiliations:** 1 Office of the Health Quarantine I Tarakan, Directorate General of Disease Control, Ministry of Health, Tarakan, Indonesia; 2 Center for The Health System and Strategy, Ministry of Health, Jakarta, Indonesia; 3 Department of Tropical Hygiene, Faculty of Tropical Medicine, Mahidol University, Bangkok, Thailand; 4 Mahidol-Oxford Tropical Medicine Research Unit, Faculty of Tropical Medicine, Mahidol University, Bangkok, Thailand; 5 Oxford University Clinical Research Unit, Jakarta, Indonesia; 6 Malaria Working Group, Directorate General of Disease Control, Ministry of Health, Jakarta, Indonesia; 7 Department of Population and Family Planning, Women’s Empowerment and Children Protection, Central Bangka, Indonesia; 8 Institute of Population and Social Research, Mahidol University, Nakhon Pathom, Thailand; 9 Centre for Tropical Medicine and Global Health, Nuffield Department of Medicine, University of Oxford, Oxford, UK; 10 The Open University, Milton Keynes, UK

**Keywords:** Malaria surveillance, Geospatial analysis, GIS, Indonesia, Health information systems, APMEN

## Abstract

As Indonesia progresses toward malaria elimination, geospatial analysis offers opportunities to strengthen surveillance, improve intervention targeting, and support data-informed decision-making. However, despite increasing availability of georeferenced surveillance data, the routine use of geographic information systems (GIS) within malaria programs remains uneven. A three-day consultation and GIS training workshop was conducted in Jakarta, bringing together 25 representatives from National and Sub-National Malaria Programs and other disease control units. The workshop included hands-on R-based spatial analysis, followed by semi-structured focus group discussions exploring current practices, barriers, and potential strategies for strengthening geospatial surveillance. Participants reported growing recognition of the value of geospatial data and described surveillance platforms that routinely capture spatially referenced information; however, these data were often used primarily for basic visualization. Key challenges included limited capacity in advanced GIS and spatial modelling, high staff turnover, data quality concerns, fragmented health information systems, and infrastructure constraints, particularly in remote settings. Participants identified opportunities to strengthen applied training and mentorship, improve data quality and interoperability across platforms, and gradually integrate geospatial analysis into routine planning and resource allocation. The consultation highlighted both progress and continuing challenges in operationalizing geospatial analysis within Indonesia’s malaria surveillance system. Continued investment in workforce capacity, data quality, infrastructure, and system integration may help strengthen the translation of routinely collected geospatial data into programmatic decision-making for malaria elimination.

**Supplementary Information:**

The online version contains supplementary material available at https://doi.org/10.1186/s12936-026-06184-5.

## Meeting background

Within the World Health Organization (WHO) Western Pacific Region, Indonesia accounted for 25.8% of malaria cases, making it the second-largest contributor after Papua New Guinea (65.6%)[1]. Nevertheless, Indonesia has made notable progress in reducing malaria incidence over the past two decades. Between 2010 and 2017, sustained elimination efforts led to a significant decline in transmission in over half of the country’s districts, covering approximately 72% of the national population [2]. However, persistent transmission in several provinces, especially in eastern regions with high population mobility, limited healthcare access, and uneven vector control coverage [3] underscores the need to continue strengthening surveillance and response strategies at both national and sub-national levels.

Timely and high-quality surveillance data are widely recognized as essential for tracking progress and informing targeted control and elimination efforts [4]. Indonesia’s malaria surveillance system, Sistem Informasi Surveilans Malaria (SISMAL), provides a national platform for case reporting and program monitoring [5]. As countries move closer to elimination, the demand for fine-scale, data-driven decision-making tends to increase [6]. Yet in many settings, limited technical capacity, fragmented systems, and restricted access to analytical software can present challenges to maximizing the utility of geospatial data.

Geographic information systems (GIS) and geospatial analysis are increasingly applied in global health, including for malaria control and elimination efforts [7–9]. Advances in open-source GIS technologies have helped reduce access barriers and support wider adoption [10]. However, the implementation of GIS within national malaria programs remains variable, including in many Asia–Pacific countries where limitations in infrastructure, technical capacity, and institutional support continue to present challenges [11].

Recognizing both opportunities and ongoing challenges in strengthening geospatial surveillance, the Asia Pacific Malaria Elimination Network (APMEN) has continued to support National Malaria Programs (NMPs) through its Surveillance and Response Working Group (SRWG). In early 2024, APMEN SRWG initiated consultations with Indonesia’s Ministry of Health to co-develop a tailored training program on GIS and geospatial analysis, in collaboration with the National and Sub-National Malaria Programs (SNMPs) and other national disease control programs. The program was technically facilitated by the Mahidol-Oxford Tropical Medicine Research Unit (MORU), host of the APMEN SRWG, and the Oxford University Clinical Research Unit Indonesia (OUCRU-ID). A series of planning meetings between March and August 2024 helped refine training priorities and ensure the use of national malaria surveillance data for practical, hands-on exercises.

The training and consultation workshop was conducted from 8–10 October 2024 in Jakarta, Indonesia. It combined lectures and hands-on R-based practical mapping such as producing choropleth maps, conducting hotspot detection, and analyzing risk factors using routine malaria data, delivered with simultaneous Bahasa Indonesia interpretation. The workshop concluded with a semi-structured focus group discussion to reflect on challenges and opportunities in applying geospatial analysis within malaria and broader disease control programs. This meeting report summarizes those discussions, outlining key constraints and potential pathways for strengthening geospatial integration. Whereas a previous APMEN meeting report examined gaps in research and capacity development for malaria surveillance and response across the Asia–Pacific [11], this report focuses on the country-level, operational use of geospatial data within Indonesia’s malaria program.

The meeting aimed to achieve the following objectives:

1. To review stakeholder-reported practices and institutional arrangements related to the use of geospatial surveillance data and analysis within the National Malaria Program and other national disease control programs.
2. To identify key barriers hindering the routine application and integration of geospatial surveillance data and analysis in malaria surveillance and programmatic decision-making.
3. To explore practical solutions and potential support mechanisms to strengthen the use of geospatial surveillance data and analysis for malaria control and elimination—particularly at sub-national levels.

## Meeting structure and process

The workshop included a series of semi-structured focus group discussions involving participants from the NMP, SNMP, and other national disease control programs, including the arboviruses unit, vector control unit, and laboratory-based disease surveillance unit. Participants were divided into three groups based on their roles in surveillance and GIS-related activities to encourage in-depth exploration of the discussion topics. Group 1 consisted of NMP and SNMP members who are public health or medical epidemiologists. Group 2 included NMP and SNMP members serving as data analytics or monitoring and evaluation officers. Group 3 comprised representatives from other national disease control programs. The discussions were facilitated in English with simultaneous interpretation in Bahasa Indonesia. Three main discussion topics were addressed in parallel by all three groups, each led by an APMEN SRWG team member. The semi-structured discussion guide was developed by the facilitator team from APMEN SRWG and OUCRU-ID. All three groups used the same semi-structured discussion guide to ensure consistency across discussions.

**Fig. 1:**
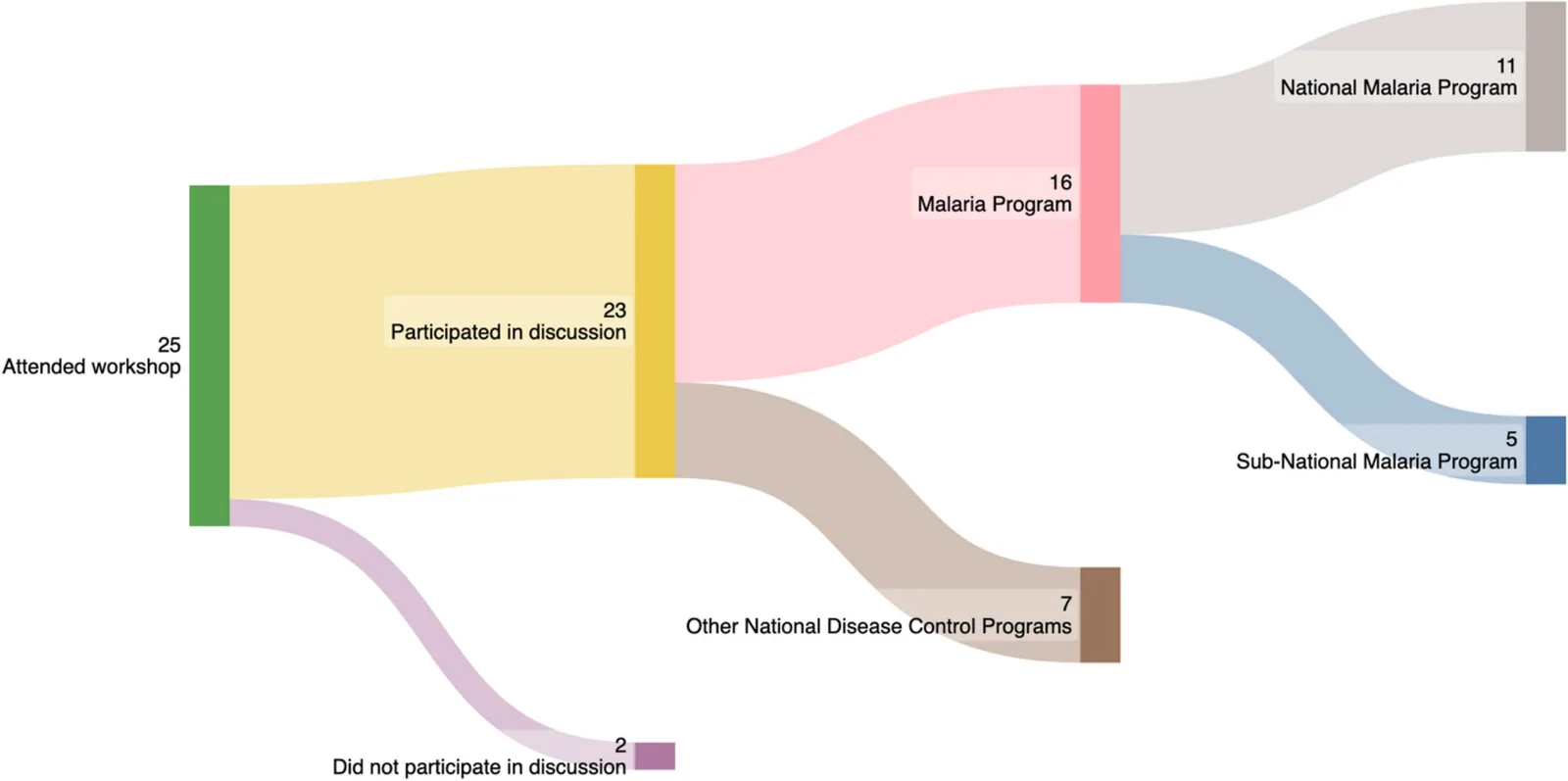
Flow of workshop participants (n = 25) and composition of the 23 discussion participants by program affiliation: National Malaria Program (n = 11), Sub-National Malaria Programs (n = 5), and other national disease control programs (n = 7)

A total of 25 participants attended the workshop, of whom 23 participated in the discussions. Among the 23 discussion participants, 16 were affiliated with the Malaria Program, comprising 11 from the National Malaria Program (NMP) and 5 from Sub-National Malaria Programs (SNMPs), while 7 were affiliated with other national disease control programs. The five SNMP representatives came from five provinces: West Papua, Papua, West Nusa Tenggara, South Sumatra, and South Sulawesi (Fig. 1).

### Topic 1. Exploration of stakeholder-reported practices on use of geospatial surveillance data and analysis

The first round of discussions focused on the following question: what are the current practices for using spatial tools and analyses in disease surveillance and control within each organization? The discussion explored the software used, types and sources of spatial data, analytical methods applied, external support received, and how geospatial findings are incorporated into decision-making processes. Participants were encouraged to reflect on methods, collaborations, and practical applications. Following a brief introduction, small-group discussions were conducted over approximately 20 min, with key points recorded on flipcharts and documented by APMEN note takers. The session concluded with short group presentations summarizing main insights, which were captured for reporting and follow-up.

### Topic 2. Identify and prioritize barriers to the use of geospatial surveillance data analysis

In this 45-min session, each participant was invited to identify barriers to applying or implementing spatial tools. The barriers were then grouped by similarity through a facilitated brainstorming process. Participants subsequently prioritized critical challenges through a brief voting exercise, with each participant selecting three barriers. The session concluded with group presentations summarizing the highest-ranked barriers, followed by a plenary reflection where facilitators synthesized common themes across groups to inform the subsequent discussion on potential solutions.

### Topic 3. Brainstorm potential solutions to address the identified barriers

The third round of discussions sought to explore participants’ ideas on how to improve the use of spatial tools and analyses for disease surveillance and control. This 45-min session began with a brief introduction and regrouping, with participants mixed into three new groups to encourage cross-program dialogue. Each participant received color-coded sticky notes indicating their affiliation (NMP, SNMP, or other national disease control programs). During a rotating station exercise, groups moved between flipchart stations every 10 min to discuss and propose solutions to the prioritized barriers, recording their suggestions on the sticky notes. The session concluded with a plenary summary, where facilitators synthesized key recommendations for further discussion and potential action.

A narrative summary approach was used to organize the information gathered from all participants. Following the workshop, facilitators from the APMEN SRWG reviewed and consolidated participants’ responses, particularly for Topics 2 and 3, and grouped them according to similarities in content, allowing common patterns and thematic categories to emerge from the participants’ responses. Findings from the three discussion groups were subsequently compared and synthesized to identify overarching themes. The final categories were developed after the workshop and represent a structured synthesis of stakeholder responses rather than a formal qualitative analysis. This paper focuses on geospatial surveillance for malaria. Findings from the discussions with participants from other disease control programs are presented in Supplementary Document S1, and Supplementary Table S1 summarizes these findings and identifies lessons relevant to the malaria program.

## Meeting findings

### Topic 1: stakeholder-reported practices on use of geospatial surveillance data analysis in the national and sub-national malaria control programs

#### Geospatial surveillance data sources

Both NMP and SNMP participants highlighted the variety of surveillance datasets available. Individual malaria case data, including travel history used to determine the importation status of cases, serves as the primary resource. To enhance malaria control efforts, additional data is collected, such as time and location of distributions of long-lasting insecticidal nets, monthly updates on identified mosquito breeding sites, and healthcare facility coordinates. Mosquito breeding site data can be used to generate receptivity maps, which serve as outputs to guide targeted interventions.

When engaged in research projects related to disease mapping led by external parties, such as research institutes and universities, they shared their own malaria data, while the external collaborators collected non-disease data, such as climate, population, and demographic information, and used it in the analysis, with results or findings shared back with the program. Participants described several key data platforms. SISMAL serves as the primary source of malaria surveillance data, collecting case data for each patient from various health facilities across Indonesia. OnaData (ONA) is currently used in certain districts to obtain geographic coordinates for specific sites, such as larval habitat. Shapefiles and demographic information are obtained from the Indonesian Central Bureau of Statistics. Participants also reported that vector surveys are carried out every six months in receptive areas to collect data on mosquito populations.

#### Geospatial platforms and types of analysis

Participants in the NMP identified Quantum GIS (QGIS) and Excel as their primary software for geospatial visualization because of simplicity and user-friendliness. Participants also reported using web-based applications such as Data wrapper for map visualization, graphic design software such as Photoshop and CorelDRAW for editing maps to reflect administrative boundary changes, and Android-based apps such as ONA and Global Positioning System (GPS) tools for collecting location coordinates.

Participants reported that the geospatial analysis methods employed by the NMP include geographic mapping of individual case distributions based on coordinates of households, as well as area-based stratification maps that depict endemicity levels based on the Annual Parasite Index (API). Additionally, the malaria program produced thematic maps, such as for visualising imported cases by collecting patient travel histories data derived from epidemiological investigation. It also developed maps of Anopheles breeding sites to identify receptive areas, along with retrospective analyses to compare malaria burdens over time.

#### Support from external partners and the use of geospatial surveillance data and analysis to guide decision making

Participants reported that GIS activities are supported by various internal and external partners of the Ministry of Health. The ministerial data centre provides server infrastructure, enabling SISMAL to operate effectively across health facilities throughout Indonesia. SISMAL, which houses all malaria-related databases, has been developed with support from the Global Fund via United Nations Development Programme (UNDP), while the United Nations Children’s Fund (UNICEF) has conducted data management training for program staff. For geospatial analysis specifically, OUCRU-ID has provided QGIS hands-on training, conducted geospatial research, and provided technical assistance to help malaria officers create their own maps, allowing the staff to share their expertise with the sub-national malaria program staff across all provinces. Additional support comes from universities, which offer training in data visualization and foster research collaborations to analyse malaria surveillance data.

Participants perceived that geospatial data and analysis are essential for planning targeted interventions, allocating resources efficiently, and supporting logistical operations. Participants described how the SISMAL V3 system enables dashboard visualizations at the national scale, displaying district-level endemicity through choropleth maps. These endemicity maps are generated using classifications based on the API, helping guide the distribution of long-lasting insecticidal nets to areas with higher malaria incidence. Additionally, maps delineating receptive areas (regions with environmental conditions conducive to malaria transmission) are used to inform vector control strategies, such as targeted larviciding in locations where mosquito breeding sites have been identified.

### Topic 2: challenges in using geospatial surveillance data and analysis in the national and sub-national malaria control programs

Focus group discussions with the NMP and SNMP identified several systemic and operational barriers to the routine application of GIS and geospatial analysis. These were grouped into six major domains. All 23 discussion participants voted, each selecting three barriers, giving a total of 69 votes (27, 21, and 21 in Groups 1, 2, and 3, respectively, as shown in Table 1).

**Table 1:** Number of votes received by each barrier, by discussion group (each participant selected three barriers)

No	Name of barriers	Number of votes		
		Group 1 (9 persons)	Group 2 (7 persons)	Group 3 (7 persons)
1	Human resource and capacity constraints	13	6	4
2	Data quality, accessibility, and integration	3	4	9
3	Infrastructure and connectivity limitations	6	2	2
4	Software and system limitations	4	1	5
5	Training and institutional support	1	7	0
6	Leadership and organizational culture	0	1	1
	Total	27	21	21

Groups 1 and 2 consisted of NMP and SNMP staff. Group 3 consisted of representatives from other national disease control programs

#### Human resource and capacity constraints

Limited technical capacity was the most frequently cited challenge raised by Malaria control program staff. Participants reported that many staff lacked the skills required for geospatial analysis, such as using QGIS, R programming, or statistical modelling techniques. In some cases, even basic computer literacy posed a barrier. Skills acquired during past GIS training were often lost due to lack of practical application in daily tasks.

Another barrier highlighted during the meeting was that high workloads and insufficient staffing further restricted the ability to carry out analysis, as geospatial work was often not integrated into core job responsibilities. Additionally, frequent staff rotation and lack of knowledge transfer between staff reduced institutional memory and disrupted continuity in geospatial data use.

#### Data quality, accessibility, and integration

Participants reported that surveillance data quality was affected by underreporting (especially from private clinics), delays in reporting, and incomplete data entry (such as blank mandatory fields in SISMAL). A lack of disaggregated data and limited access to auxiliary datasets, including population maps, climate indicators, and updated shapefiles, limited the potential for advanced geospatial modelling.

#### Infrastructure and connectivity limitations

Participants also reported that poor internet connectivity and limited hardware availability, including personal computers and GPS devices, were particularly problematic in remote and high-burden provinces such as the Papua region, which participants noted accounts for over 95% of reported malaria cases nationally. Since geospatial analysis and tools often depend on internet access for cloud-based tools and data visualization, these limitations significantly hindered workflows.

#### Software and system limitations

Although QGIS was frequently used because it is open-source, some participants found it unintuitive and difficult to use, especially for those without prior technical backgrounds. There was also limited access to licensed alternatives, as financial resources for proprietary GIS software were generally unavailable. While free GIS programs were preferred, their perceived complexity posed challenges for broader uptake, particularly among district health offices and primary health centre staff.

#### Training and institutional support

Participants reported limited training opportunities, and even when training was available, it often focused only on basic mapping rather than advanced geospatial analysis. There was a strong call for continuing education, hands-on mentorship, and modular training in Bahasa Indonesia. Limited knowledge-sharing between staff further hindered skills development.

#### Leadership and organizational culture

Participants noted that in districts that had achieved malaria elimination, malaria was no longer prioritized, leading to reduced funding for the malaria program.

### Topic 3: needs and support to strengthen the use of geospatial surveillance data analysis in national and sub-national malaria control programs

#### Strengthening analytical skills and institutional collaboration

To address challenges related to basic computer literacy, limited opportunities to apply GIS in routine work, and the retention of skills amid staff rotation, participants proposed several strategies, including in-person and online training, on-the-job mentoring, and integration of geospatial analysis into routine responsibilities. Collaboration with academic institutions and research partners was suggested to support sustained skills development and applied research opportunities.

#### Streamlining data reporting and improving quality assurance

According to the participants’ views, the effectiveness of geospatial analysis was seen to depend on the quality of surveillance data stored in SISMAL. Participants recommended simplifying reporting forms, clarifying required variables, and conducting routine validation audits. Private sector engagement was also viewed as critical, with suggestions to link data submission to licensing incentives or performance recognition.

#### Enhancing infrastructure and connectivity for optimal GIS operations

Most attendees believed that in remote or high-burden areas with limited access to functioning computers, investments in basic hardware and expansion of internet access (e.g. satellite-enabled solutions) would improve reporting flexibility and GIS use in the field, in addition to the improvement of mobile data entry platforms.

#### Facilitating access to user-friendly GIS tools for routine mapping

QGIS was commonly used due to its free access, although it was perceived as challenging for beginners. Participants requested simpler interfaces, improved user documentation, and embedded templates for routine mapping tasks.

#### Targeted GIS training and leadership-supported capacity building

Training opportunities in GIS and geospatial analysis were seen as ad hoc, too brief, and often limited to introductory content. Participants called for more modular, hands-on training, particularly in Bahasa Indonesia, covering basic and advanced methods. They also highlighted the need for post-training support and structured peer learning mechanisms within malaria programs. In some sub-national areas, addressing institutional barriers of lack of leadership prioritization and reduced focus on malaria after elimination certification were noted as necessary to sustain GIS capacity. Participants highlighted the importance of fostering a leadership mindset to ensure continued prioritization of capacity-building programs, even after an area has been certified malaria-free.

## Discussion

This paper presents insights from stakeholder focus group discussions during a national training workshop on GIS and geospatial analysis. It was designed to explore the use of surveillance data and the implementation of geospatial analysis within Indonesia’s malaria surveillance system. Participants from the national malaria program, sub-national malaria programs, and other disease control units identified key opportunities, barriers, and priorities for more effectively integrating geospatial analysis into routine public health programming.

### Progress and current use of geospatial surveillance data and analysis platforms

Participants across national and sub-national levels expressed increasing recognition of the utility of geospatial data for enhancing malaria surveillance and supporting targeted interventions. National disease surveillance systems such as SISMAL, SILANTOR (vector control information system), and SIARVI (arbovirus information system) routinely collect georeferenced information, including the detailed addresses of cases, entomological surveys, and health facilities. However, these data are generally underutilized beyond basic visualization. More advanced geospatial analyses (such as hotspot detection, spatiotemporal clustering, and ecological modeling) remain infrequent in operational workflows.

This pattern of underuse reflects systemic challenges documented in low- and middle-income countries (LMICs), where the analytical capacity needed to convert granular geospatial data into actionable insights remains limited [12, 13]. Geospatial data infrastructure has been recognized as a foundational element in supporting data-driven public health systems by promoting interoperability, standardization, and timely decision-making [14]. However, persistent barriers, such as regional disparities in infrastructure, regulatory and structural fragmentation, integration difficulties with legacy systems, and limited financial investment, continue to hinder the development of effective spatial data infrastructure in many parts of Africa and Asia [14–16].

### Barriers to implementation of GIS and geospatial analysis

Stakeholder discussions identified multiple interconnected barriers that continue to constrain the routine application of GIS and geospatial analysis within Indonesia’s malaria surveillance system. Although SISMAL has strengthened national data reporting, participants emphasized the need to further expand its functionality to better support routine spatial analysis and decision-making, consistent with earlier calls to integrate geospatial intelligence into malaria surveillance [5, 17]. One potential enhancement to SISMAL for spatial analysis would be the ability to perform automated hotspot or cluster analysis, which could automate complex analytical techniques and help the NMP and SNMPs explore spatial patterns in malaria data.

Infrastructure limitations were also frequently cited (8 of 48 votes from malaria program staff; Table 1), particularly in remote and high-burden districts. Uneven access to functioning hardware, limited server capacity, and unreliable internet connectivity were reported to restrict the effective use of GIS platforms. Such constraints mirror experiences in other parts of South and Southeast Asia, where digital infrastructure gaps continue to limit the operationalization of geospatial tools in public health settings [18, 19].

Data-related challenges were also prominent. Participants raised concerns regarding data completeness, timeliness, and geolocation accuracy, as well as the limited availability of high-resolution spatial datasets and updated administrative boundary shapefiles. Inconsistent data standards and variable data quality across districts were perceived as significant barriers to conducting reliable spatial analysis and advanced modeling. These challenges highlight the foundational importance of strengthening data governance and quality assurance mechanisms to enable meaningful geospatial insights.

Changes in administrative boundaries over time, such as the establishment of new provinces in the Papua region in 2022, also require version-controlled boundary datasets so that malaria indicators can be compared consistently across periods. As the use of household-level coordinates expands, governance arrangements should also address the protection of location data, for example through role-based access and the aggregation or masking of locations displayed on public dashboards. It is also important to distinguish basic cartographic visualization, such as choropleth mapping, from formal spatial analyses such as hotspot detection and spatiotemporal modeling, which require different competencies and governance arrangements.

Technical capacity gaps further constrained implementation. Many health officers reported limited exposure to analytical tools such as QGIS or R, minimal experience in spatial data management, and insufficient training in advanced geospatial and statistical modeling. High staff turnover and the absence of structured mechanisms for skills retention were noted to exacerbate these challenges. In some settings, gaps in basic digital literacy were reported, underscoring the need to pair advanced geospatial training with foundational digital competencies. Similar barriers have been documented in other LMIC contexts, where limited mentorship opportunities and under-resourced training systems have hindered the institutionalization of geospatial competencies within routine surveillance systems [20, 21].

Fragmentation of health information systems represented another recurring concern. Disease-specific databases, including those for malaria, vector-borne diseases, and syndromic surveillance, often operate independently with limited interoperability or harmonization of data structures. Although the Ministry of Health has introduced the national SATUSEHAT platform to enhance interoperability across health information systems, integration remains ongoing. Several programs have initiated data linkages or are transitioning to the platform, including the tuberculosis information system and medical equipment information systems, while many program-specific systems continue to operate in parallel. Furthermore, integrating malaria case data from SISMAL with vector surveillance data from SILANTOR could enable spatial analyses that link epidemiological and entomological information. Integrating both data sources at comparable spatial and temporal scales could support more informed malaria risk stratification.

This fragmentation restricts opportunities for integrated geospatial analyses that could better inform cross-program planning in areas with overlapping disease burdens. Such system-level challenges have been widely reported in global health information system assessments [18, 22, 23]. Participants suggested that gradual efforts to harmonize data elements, improve interoperability, and strengthen linkages between key platforms (such as SISMAL and SILANTOR) could help support more integrated and spatially informed decision-making over time.

### Opportunities for strengthening and institutionalizing GIS capacity

Despite the identified barriers, participants highlighted several opportunities for advancing GIS integration, particularly for the malaria program. Participants noted that malaria programs could build on experiences from other infectious disease programs in Indonesia, where GIS and spatiotemporal modeling have been applied [24–26]. Tools such as real-time dashboards within the SKDR (early warning and response) system (Supplementary Document S1) and cross-program data integration were identified as promising models to enhance geospatial intelligence. There was strong interest in embedding GIS into routine planning, monitoring, and resource allocation processes. While initial implementation has been supported by donors and academic partners, participants noted the importance of progressively institutionalizing GIS platforms within national and sub-national workflows to promote long-term sustainability. This approach is consistent with experiences from other countries, where integrating geospatial decision-support systems into health strategies has contributed to improved efficiency, responsiveness, and equity in service delivery [27, 28].

Participants also underscored the importance of moving beyond ad hoc training. Sustainable capacity development will require structured mentorship, applied analysis using routine program data, and ongoing supervision and peer learning. Evidence from LMIC settings demonstrates that long-term engagement and iterative practice strengthen technical performance and promote data-informed decision-making [29–31]. The development of modular, language-appropriate training materials (particularly in Bahasa Indonesia) was viewed as important to facilitate implementation across decentralized levels. In addition, partnerships with universities and technical institutions could help support certified training and in-service learning programs, contributing over time to a more sustainable cadre of geospatial analysts and strengthening geospatial infrastructure for malaria elimination [32, 33].

## Limitations

This report has several limitations. First, as participants were not selected for national representativeness, the findings reflect the perspectives of workshop participants and may not fully represent surveillance practices across Indonesia. Second, the needs identified and areas of support proposed during the workshop should be considered preliminary. Further assessment is warranted to refine these proposals through policy and document review and broader stakeholder consultation to establish measurable implementation indicators and determine implementation timeframes. Third, findings were derived from facilitator notes and flipcharts rather than verbatim transcripts, and some meaning may have been lost through simultaneous interpretation. Fourth, the workshop was facilitated and reported by the same APMEN-linked team in a Ministry-supported setting, which may have introduced social-desirability bias or reluctance to criticize existing systems. Finally, the effectiveness of the GIS training and any subsequent implementation were not assessed.

## Recommendation summary

Several strategic priorities emerged from stakeholder discussions to support the effective and sustainable use of geospatial analysis:

1. Strengthening human resource capacity in geospatial analysis and statistical modelling through development of flexible modular learning resources and structured mentorship schemes to support skills retention, especially for high staff turnover settings.
2. Improving data quality and accessibility through standardizing data entry, regular data audits, and validation tools in order to support continuous data quality improvement and encourage timely reporting
3. Enabling system integration and interoperability across platforms to better link disease-specific surveillance systems and reduce fragmentation
4. Addressing infrastructure and connectivity constraints by expanding reliable internet access and ensuring equitable access to appropriate hardware, software, and GPS-enabled devices
5. Institutionalizing geospatial analysis in routine decision-making through accessible dashboards and decision-support tools, supported by strong leadership and collaboration with universities, research institutes, and technical organizations.

## Conclusions

This national stakeholder consultation identified perceived gaps in the routine use of geospatial information within Indonesia’s malaria surveillance program, particularly in workforce capacity, data quality, interoperability, infrastructure, and integration of spatial analysis into program workflows. The findings reflect the perspectives of workshop participants and should not be interpreted as a representative assessment of all Indonesian districts. A phased approach combining standardized geospatial data governance, routine analytical products, mentorship, interoperable systems, and measurable implementation indicators may help translate routinely collected spatial information into malaria program action. Future work should evaluate whether these measures improve data quality, timeliness of response, intervention targeting, and prevention of malaria re-establishment.

## Supplementary Information

Supplementary Material 1.

## Data Availability

No datasets were generated or analysed during the current study.

## Abbreviations

API: Annual Parasite Index
APMEN: Asia Pacific Malaria Elimination Network
DFAT: Department of Foreign Affairs and Trade
GIS: Geographic information system
GPS: Global Positioning System
LMIC: Low- and middle-income country
MORU: Mahidol-Oxford Tropical Medicine Research Unit
NMP: National Malaria Program
ONA: OnaData
OUCRU-ID: Oxford University Clinical Research Unit Indonesia
QGIS: Quantum GIS
SIARVI: Sistem informasi arbovirosis
SILANTOR: Sistem Informasi Surveilans Vektor
SISMAL: Sistem Informasi Surveilans Malaria
SKDR: Sistem Kewaspadaan Dini dan Respon
SNMP: Sub-National Malaria Program
SRWG: Surveillance and Response Working Group
UNDP: United Nations Development Programme
UNICEF: United Nations Children's Fund
WHO: World Health Organization

## References

[1] World Health Organization, World malaria report 2025: addressing the threat of antimalarial drug resistance. 2025.

[2] Sitohang V, et al. Malaria elimination in Indonesia: halfway there. Lancet Glob Health. 2018;6(6):e604–6. 10.1016/S2214-109X(18)30198-0 29703663

[3] Herdiana H, et al. Shrinking the malaria map in Indonesia: progress of subnational control, elimination, and future strategies. BMC Med. 2025;23(1):512. 10.1186/s12916-025-04355-w PMC12403376 40890766

[4] Ohiri K, et al. An assessment of data availability, quality, and use in malaria program decision making in Nigeria. Health Syst Reform. 2016;2(4):319–30. 10.1080/23288604.2016.1234864 31514720

[5] Asmiani A, Windusari Y, Hasyim H. Malaria vector control and the Electronic Malaria Surveillance Information System (E-SISMAL) in Bangka Barat Regency Indonesia. Jurnal Kesehatan Lingkungan. 2021;13(4):241–9.

[6] Kang SY, et al. Fine-scale maps of malaria incidence to inform risk stratification in Laos. Malar J. 2024;23(1):196. 10.1186/s12936-024-05007-9 PMC11202256 38918779

[7] Odhiambo JN, et al. Spatial and spatio-temporal methods for mapping malaria risk: a systematic review. BMJ Glob Health. 2020;5(10):e002919. 10.1136/bmjgh-2020-002919 PMC7537142 33023880

[8] Rotejanaprasert C, et al. Spatiotemporal patterns and association with climate for malaria elimination in Lao PDR: a hierarchical modelling analysis with two-step Bayesian model selection. Malar J. 2024;23(1):231. 10.1186/s12936-024-05064-0 PMC11298089 39098946

[9] Rotejanaprasert C, et al. Bayesian spatio-temporal distributed lag modeling for delayed climatic effects on sparse malaria incidence data. BMC Med Res Methodol. 2021;21:1–15. 10.1186/s12874-021-01480-x PMC8690908 34930128

[10] Barlow S, Spencer J, Wilkes B, Innovations in geographic information systems mapping technology: GIS Working Group Meeting, MEASURE Evaluation, University of North Carolina at Chapel Hill. 2017.

[11] Sirimatayanant M, et al. Gaps in research and capacity development for malaria surveillance and response in the Asia–Pacific: meeting report. Malar J. 2023;22(1):91. 10.1186/s12936-023-04459-9 PMC10000341 36899358

[12] Tanser FC, Le Sueur D. The application of geographical information systems to important public health problems in Africa. Int J Health Geogr. 2002;1:1–9. 10.1186/1476-072X-1-4 PMC149399 12537589

[13] Mennecke BE, West LA Jr. Geographic information systems in developing countries: issues in data collection, implementation and management. J Glob Inf Manag (JGIM). 2001;9(4):44–54.

[14] Adeleye VM, et al. Advancing public health through spatial data infrastructures: a review of global practices, governance and policy recommendations. Discov Public Health. 2025;22(1):1–11.

[15] Makanga P, Smit J. A review of the status of spatial data infrastructure implementation in Africa. S Afr Comput J. 2010;2010(45):18–25.

[16] Hoxha K, et al. Understanding the challenges associated with the use of data from routine health information systems in low-and middle-income countries: a systematic review. Health Inf Manag J. 2022;51(3):135–48. 10.1177/1833358320928729 32602368

[17] Zainuddin Z, Hendrati LY. Evaluation of malaria surveillance system in department of health district Sumbawa Besar. Jurnal Berkala Epidemiologi. 2014;2(3):342–54.

[18] Rahi M, Sharma A. For malaria elimination India needs a platform for data integration. BMJ Glob Health. 2020;5(12):e004198. 10.1136/bmjgh-2020-004198 PMC7780526 33380414

[19] Rotejanaprasert C, et al. Malaria epidemiology, surveillance and response for elimination in Lao PDR. Infect Dis Poverty. 2024;13(1):35. 10.1186/s40249-024-01202-7 PMC11112833 38783374

[20] Larocca A, Moro Visconti R, Marconi M. Malaria diagnosis and mapping with m-Health and geographic information systems (GIS): evidence from Uganda. Malar J. 2016;15:1–12. 10.1186/s12936-016-1546-5 PMC5075756 27776516

[21] Ali A, et al. Factors impeding the implementation of spatial data infrastructure in lower middle-income economies: a case study from Pakistan. Inf Dev. 2025. 10.1177/02666669241302788.

[22] Merkord CL, et al. Integrating malaria surveillance with climate data for outbreak detection and forecasting: the EPIDEMIA system. Malar J. 2017;16(1):89. 10.1186/s12936-017-1735-x PMC5324298 28231803

[23] Al Manir MS, et al. A surveillance infrastructure for malaria analytics: provisioning data access and preservation of interoperability. JMIR Public Health Surveill. 2018;4(2):e10218. 10.2196/10218 PMC6026300 29907554

[24] Ramadona AL, et al. Predicting the dengue cluster outbreak dynamics in Yogyakarta, Indonesia: a modelling study. Lancet Reg Health Southeast Asia. 2023;15:100209. 10.1016/j.lansea.2023.100209 PMC10442971 37614350

[25] Farkhan A, et al. Spatiotemporal epidemiology and associated risk factors of tuberculosis incidence and mortality in Indonesia 2017–2022: a nationwide space-time hierarchical analysis. Popul Health Metr. 2026;24:12. 10.1186/s12963-026-00458-5 PMC12952012 41620746

[26] Farkhan A, et al. Spatiotemporal epidemiology, geographic hotspots, and risk factor associations of drug-resistant tuberculosis incidence in Indonesia: a Bayesian hierarchical modelling approach. Infect Dis Poverty. 2026;15(1):23. 10.1186/s40249-026-01418-9 PMC12903336 41680935

[27] Kelly GC, et al. Malaria elimination: moving forward with spatial decision support systems. Trends Parasitol. 2012;28(7):297–304. 10.1016/j.pt.2012.04.002 22607693

[28] Kirk KE, et al. Geospatial technology: a tool to aid in the elimination of malaria in Bangladesh. ISPRS Int J Geo-Inf. 2014;4(1):47–58.

[29] Rotejanaprasert C, et al. Perspectives and challenges in developing and implementing integrated dengue surveillance tools and technology in Thailand: a qualitative study. PLoS Negl Trop Dis. 2024;18(8):e0012387. 10.1371/journal.pntd.0012387 PMC11324148 39141623

[30] Blanford JI. Managing vector-borne diseases in a geoAI-enabled society. Malaria as an example. Acta Trop. 2024;260:107406. 10.1016/j.actatropica.2024.107406 39299478

[31] Sipe NG, Dale P. Challenges in using geographic information systems (GIS) to understand and control malaria in Indonesia. Malar J. 2003;2(1):36. 10.1186/1475-2875-2-36 PMC305351 14613511

[32] Sjoukema J-W, et al. Governance interactions of spatial data infrastructures: an agent-based modelling approach. Int J Digit Earth. 2021;14(6):696–713.

[33] Mutale W, et al. Improving health information systems for decision making across five sub-Saharan African countries: implementation strategies from the African Health Initiative. BMC Health Serv Res. 2013;13(2):S9. 10.1186/1472-6963-13-S2-S9 PMC3668230 23819699

