# Supplementary material for: Strengthening geospatial surveillance for malaria control and elimination in Indonesia: a meeting report
Source: Malar J. 2026 Oct 13;25:363. doi: 10.1186/s12936-026-06184-5 (PMC13660323; doi:10.1186/s12936-026-06184-5)
Supplement: Supplementary file 1 [file 12936_2026_6184_MOESM1_ESM.docx]

**Strengthening geospatial surveillance for malaria control and elimination in Indonesia: a meeting report**

**Supplementary Document S1: Geospatial surveillance practices, challenges, and needs in other disease control programs**

1. **Stakeholder-reported practices in other disease control programs**

This supplementary document summarizes information reported by participants from non-malaria disease control programs during the workshop; system descriptions reflect stakeholder-reported practices and were not independently verified against official documentation. Participants reported that, similar to the malaria program, other disease control programs maintain their own information systems that incorporate spatial displays into their dashboards.

a. **Geospatial surveillance data sources**

A wide variety of geospatial data is utilized across national disease control programs in Indonesia, with each system tailored to meet the specific surveillance needs of different health conditions. The Vector Surveillance Information System, or *Sistem Informasi Surveilans Vektor* (SILANTOR), collects spatial data related to dengue vector density, malaria vector receptivity, and insecticide resistance, supporting vector control activities. The laboratory surveillance working group gathers geospatially tagged information through stock reports and outbreak investigations, which are then integrated into the national laboratory monitoring and evaluation system.

The *Sistem Informasi Arbovirosis* (SIARVI) or Arboviruses Information System manages case-based data, mortality figures, and logistical information related to arbovirus outbreaks such as dengue and chikungunya. Meanwhile, the *Sistem Kewaspadaan Dini dan Respon* (SKDR) functions as an early warning and response system, capturing geospatial patterns of 25 symptom-based disease conditions. This enables rapid detection of potential outbreaks and facilitates timely response across districts and provinces. Lastly, the New All Records (NAR) platform stores spatial data on laboratory-confirmed and negative cases of Mpox, COVID-19, influenza-like illness, severe acute respiratory infections, and other emerging diseases.

These data are primarily sourced through routine surveillance and reporting activities conducted at the facility level. SILANTOR relies on monthly reports submitted by primary healthcare centers (PHCs), particularly for field activities such as larval surveys, rodent trapping, and entomological monitoring. It also incorporates data from regional health laboratories, which contribute monitoring and evaluation inputs. SIARVI receives case and logistics data directly from PHCs, while SKDR aggregates reports from both PHCs and hospitals. Most systems follow a bottom-up reporting structure, where data are transmitted from district health offices (DHOs) to the provincial and national levels. DHOs consolidate data from the PHCs in their jurisdiction, enabling spatially disaggregated reporting and more localized response planning.

b. **Geospatial platforms and types of analysis**

Each national disease control program operates its own dedicated, web-based geospatial surveillance system tailored to its specific disease area. These platforms are used for case reporting, monitoring, evaluation, and resource planning. They are accessible online to health facilities and laboratories across Indonesia and ensure that case reporting adheres to national disease-specific protocols.

The arbovirus program utilizes QGIS to visualize spatial data for diseases such as dengue, chikungunya, and Japanese encephalitis. This geospatial analysis is based on case, mortality, and logistics data stored in the SIARVI platform (https://siarvi.kemkes.go.id/). Meanwhile, the vector control program manages and visualizes data through SILANTOR (https://silantor.kemkes.go.id/), which provides spatial insights into vector densities, receptivity, and insecticide resistance. The syndromic surveillance program uses the SKDR platform (https://skdr.surveilans.org/auth) to detect early warning signals based on spatial patterns of 25 notifiable syndromes prior to laboratory confirmation. Lastly, the laboratory-based national surveillance program manages the NAR system (https://allrecord-tc19.kemkes.go.id/), which maps and stores individual case-level data for confirmed and negative cases of COVID-19, acute hepatitis, Mpox, and other emerging infections.

Each program applies tailored analytical tools based on its surveillance goals. SILANTOR integrates with Google Data Studio to generate spatial maps of vector density indicators, supporting localized vector control planning. SKDR produces spatial overlays of high-risk areas and conducts temporal trend analysis to monitor potential outbreaks in real time based on syndromic data. NAR provides similar visualizations but focuses on laboratory-confirmed cases, supporting epidemiological investigation and diagnostic-based disease monitoring. As described by participants, most of these functions involve cartographic visualization (e.g., thematic maps, dashboards, and spatial overlays) rather than formal spatial statistical analysis such as cluster detection or spatiotemporal modeling.

c. **Support from external partners** **and the use of geospatial surveillance data and analysis to guide decision-making**

External support for data analysis, particularly for SKDR, is provided by the World Health Organization (WHO) country office and the Public Health Emergency Operations Center, which assist in enhancing analytical capacity and response coordination. The participants mentioned that results from geospatial analyses can play a crucial role in decision-making across various disease surveillance systems. Geospatial analysis in SKDR supports early warning of potential outbreaks at the district level, which can help plan public health responses and case management. Similarly, SILANTOR and SIARVI use geospatial analysis to enhance early detection and guide interventions for diseases such as dengue, malaria, and leptospirosis. SKDR and SIARVI enable public health measures such as health education and epidemiological investigation; transport and laboratory analysis of collected specimens; and case management including quarantine and isolation. Participants recognized that vector control and rodent control driven by geospatial data can be useful for targeting resources for larviciding, fogging, and deploying rodent traps in high-risk areas.

2. **Challenges experienced in other control programs**

Participants from other disease control units, including vector-borne diseases and syndromic surveillance, reported a series of barriers similar to those faced by malaria programs, with some additional cross-sectoral concerns. These were grouped into six key domains.

a. **Human resource and capacity constraints**

Other disease control programs also reported a lack of personnel trained in GIS and geospatial methods. Staff were typically focused on data reporting rather than analysis, and geospatial skills were rarely embedded in job descriptions. As with the malaria program, routine opportunities for geospatial analysis were limited, leading to underuse of available datasets.

b. **Data quality, accessibility, and integration**

Fragmentation of health information systems emerged as a major issue raised by participants. Programs often maintained separate reporting platforms with incompatible variables and structures, making integration difficult. Participants noted that, although dashboards display basic maps, most systems lacked automated spatial analysis functions, so more detailed mapping required manual data extraction.

In addition to system fragmentation, data completeness and reporting consistency were identified as ongoing challenges among the stakeholders. For example, health facilities and laboratories lacked system access or user credentials for certain disease reporting platforms, contributing to incomplete data streams. Inconsistent routine reporting further impaired data timeliness, undermining efforts to perform real-time geospatial analyses. These issues reflect broader concerns about surveillance data quality, especially in resource-limited settings.

c. **Infrastructure and connectivity limitations**

A common issue from participants’ experience was unreliable internet and limited access to devices, especially in rural and underserved areas. These issues constrained access to web-based platforms and hindered real-time data analysis. Participants perceived these challenges to be most pronounced in areas with the highest disease burden.

d. **Software and system limitations**

Several software-related barriers were noted during the discussion. Concerns about system security, including risks of hacking and unauthorized access, were raised in relation to both local and web-based platforms. Limited server capacity and frequent downtime were reported to disrupt workflows and reduce system reliability. Participants also described the overwhelming proliferation of disease-specific information systems, which has created redundancies and inefficiencies. This fragmentation is partly rooted in long-standing institutional norms, with each disease control unit developing and maintaining its own bespoke system.

e. **Training and institutional support**

Similar to malaria programs, other units reported limited access to structured GIS training. When training did occur, it was often short and focused only on software operation rather than application to public health decision-making. Additionally, there were few institutional mechanisms for follow-up or reinforcement of skills.

f. **Leadership and organizational culture**

Participants described a culture of limited communication around analytical work. Staff were often hesitant to ask questions or share difficulties, which limited opportunities for collaborative learning. A more supportive environment with mechanisms for mentorship, recognition, and knowledge exchange was seen as necessary to advance geospatial analytical capacity across disease programs.

3. **Needs and support for enhancing the use of surveillance data and geospatial analysis in other disease control programs**

Participants representing non-malaria disease control programs identified several critical enablers needed to improve the implementation and routine use of geospatial analysis in their respective domains.

a. **Addressing workforce gaps through targeted recruitment, capacity-building, and collaboration**

Other disease control units reported critical workforce shortages, with many staff focused on routine data reporting and having limited exposure to GIS methods. Participants proposed targeted recruitment, on-the-job training, and staff sharing across programs to address short-term gaps. Strengthening partnerships with universities and NGOs could support longer-term capacity-building. Such collaborations could facilitate applied research projects that continuously enhance technical expertise in GIS.

b. **Building an interoperable disease surveillance system**

Fragmented disease information systems were a recurring concern. Disparate platforms (e.g. SIARVI, SILANTOR) operate independently with inconsistent variable definitions and limited interoperability. Participants proposed harmonizing surveillance systems via initiatives like the national “Satu Sehat” platform and bridging data between disease programs.

They emphasized that the platform could enable smoother data exchange and help reduce duplication of reporting efforts. They also highlighted that the malaria program could simplify data fields to improve usability and integration, focusing on essential indicators while ensuring that critical information remains available and useful for decision-making.

c. **Improving internet infrastructure and offline-capable data collection**

Poor internet connectivity and limited hardware access in remote districts were identified as barriers that hindered real-time data entry and use of web-based GIS platforms. Participants emphasized the need for improved digital infrastructure and suggested hybrid online and offline systems to enable flexibility in data collection and visualization.

d. **Improving data management software systems**

Frequent system downtimes and concerns over data security were seen as obstacles to streamlined data use. Participants proposed improving server capacity to enhance system stability with minimal disruptions and prevent data loss. In addition, enhancing platform usability through intuitive design and user feedback would make the system easier to navigate and more responsive to users’ operational needs.

e. **Enhancing access to GIS capacity for programmatic decision-making**

Noting that limited geospatial training restricted their exposure to GIS, stakeholders called for practical training sessions on mapping basics and on interpreting geospatial dashboards for program action. Participants also highlighted the need for institutional encouragement and follow-up support to sustain the improved skills within the organization.

f. **Strengthening organizational culture and peer recognition for GIS data use**

Participants noted that organizational culture strongly influences the use of geospatial data, with poor internal communication, reluctance to share challenges, and limited cross-program dialogue acting as barriers to innovation. Building a more collaborative environment with incentives for performance and peer recognition was viewed as necessary for advancing geospatial analysis across programs.

| **Program** | **Other disease control programs** | | | | **Relevant lesson for malaria** |
| --- | --- | --- | --- | --- | --- |
|  | **Vector control** | **Arboviruses** | **Early warning program** | **Laboratory-confirmed surveillance** |  |
| **Geospatial surveillance data** | Dengue vector density, malaria vector receptivity, and insecticide resistance | Case-based data, mortality figures, and logistical information on arbovirus outbreaks such as dengue and chikungunya | Individual and aggregate data on 25 notifiable symptom-based disease conditions prior to laboratory confirmation | Individual-level laboratory-confirmed case data for diseases such as Mpox, COVID-19, influenza-like illness, acute hepatitis, and severe acute respiratory infections | As in other programs, malaria geospatial mapping relies on individual case data that can be aggregated by geographic area. Linking SISMAL case data with SILANTOR entomological data (e.g., vector density and insecticide resistance) at comparable spatial and temporal scales could support malaria receptivity assessment and stratification. |
| **Geospatial platforms and types of analysis** | Vector Surveillance Information System (SILANTOR). The link can be accessed in <https://silantor.kemkes.go.id/>  SILANTOR provides a publicly accessible dashboard, while access to some data is restricted to authorized staff through login credentials.  The system has been integrated with Google Data Studio to generate spatial maps of vector density indicators. | Arboviruses Information System (SIARVI). The link can be accessed in <https://siarvi.kemkes.go.id/>  Access to SIARVI is restricted to authorized staff.  The system provides spatial maps that highlight arboviral incidence and mortality rates across specific areas. | Early warning surveillance information system (SKDR). The link can be accessed in <https://skdr.surveilans.org/auth>  Access to the SKDR is restricted to authorized staff.  The system's dashboard displays spatial information on 25 notifiable syndromes as early warning signals of potential outbreaks. | New All Records (NAR). The link can be accessed in <https://allrecord-tc19.kemkes.go.id/>  Access to the NAR is restricted to authorized staff.  NAR provides similar visualizations to support epidemiological investigation and diagnostic-based disease monitoring. | The malaria program uses SISMAL to visualize spatial information, while other tools, such as Excel and QGIS, are also utilized for reporting the current malaria situation. Access to SISMAL is restricted to authorized staff. SILANTOR's combination of a public dashboard with credential-restricted data, and SKDR's dashboard-based early warning signals, could inform routine SISMAL outputs, such as automated maps of cases and foci and early alerts for case increases, while protecting household-level locations. |

| **upport from external partners** **and the use of geospatial surveillance data and analysis to guide decision-making** | Geospatial analysis used to enhance early detection and guide interventions | Geospatial analysis used to enhance early detection and guide interventions | World Health Organization (WHO) through the Public Health Emergency Operations Center.  Geospatial analysis used to enhance early detection and help epidemiological investigation. | Geospatial analysis used to enhance early detection and guide interventions |  |
| --- | --- | --- | --- | --- | --- |

**Table S1.** Summary of stakeholder-reported geospatial surveillance practices in non-malaria disease control programs in Indonesia and relevant lessons for the malaria program.
